# The ongoing impact of the Covid-19 pandemic on endometriosis patients: A survey of 1,089 UK patients

**DOI:** 10.52054/FVVO.14.3.037

**Published:** 2022-09-30

**Authors:** J.I. Spencer, G Mezquita, F Shakir

**Affiliations:** Royal Free Hospital, Pond St, London, NW3 2QG; University College London Hospital, 235 Euston Rd, London, NW1 2BU

**Keywords:** Covid-19, Endometriosis, Mental Health

## Abstract

**Background:**

The impact of Covid-19 on endometriosis patients is under-researched. Endometriosis has significant psychosocial effects on patients. Moreover, the mainstay of diagnosis and treatment of endometriosis is elective surgery, impacted as a result of healthcare strain.

**Objective:**

To better understand the effect of the Covid-19 pandemic on endometriosis patients

**Materials and Methods:**

An online survey sent to adult UK endometriosis patients between 27th August and 15th September 2021. The study received HRA and HCRW research ethic committee approval.

**Main outcome measures:**

Effects of the Covid-19 pandemic on endometriosis symptoms and surgery

**Results:**

We received 1,089 survey responses. Respondents had a median age of 34, and 82.0% of respondents were white British. 18.8% of respondents reported a previous positive Covid-19 PCR test. 84.6% of patients had been double vaccinated at time of response. 20 patients reported Covid-related hospital admission, with 1 requiring intubation. Large numbers of patients (31.4-55.2%) reported worsening of endometriosis symptoms during the pandemic. 69.2% of respondents reported worsening of associated mental health symptoms. Whilst 44% of respondents had elective endometriosis surgery planned, the majority of operations were disrupted, and 18.7% of total respondents did not have a new surgery date.

**Conclusions:**

More research and support are needed for endometriosis patients as they wait longer for surgery. A holistic approach, encompassing mental health needs, may be particularly beneficial for patients.

**What is new?:**

This is the first survey examining the effects of Covid-19 on endometriosis patients including data beyond January 2021.

## Introduction

Endometriosis affects 5-10% of women of reproductive age (Taylor et al., 2021), a similar prevalence to diabetes mellitus. There is an enormous associated healthcare cost and impact on patients’ quality of life (Simoens et al., 2012).

There is limited research into the impact of Covid-19 on endometriosis, which is striking in comparison to diseases of similar prevalence. For example, at the time of writing, a PubMed search for “endometriosis” and “Covid-19” yields 42 results, compared to 10,635 results for “diabetes” and “Covid-19”. There are potential biological interactions, since endometriosis has systemic inflammatory effects and can affect the thorax (Taylor et al., 2021). Effects of the pandemic may be more pertinent. Endometriosis is associated with anxiety and depression, which significantly impact patients’ quality of life (Laganà et al., 2017). Moreover, the gold-standard method of diagnosis and treatment of endometriosis is surgery, which has been significantly disrupted (Søreide et al., 2020).

There are only 14 published studies examining the effects of Covid-19 on endometriosis patients (Arena et al., 2021; Armour et al., 2022; Ashkenazi et al., 2022; Barretta et al., 2022; Demetriou et al., 2021; Evans et al., 2021; Moazzami et al., 2021; Nicolás et al., 2022; Rosielle et al., 2021; Schwab et al., 2021; Schwab et al., 2022a; Schwab et al., 2022b; Uccella et al., 2021; Yalçın Bahat et al., 2020). Of these, three published papers appear to have analysed results from the same survey (Schwab et al., 2021; Schwab et al., 2022a; Schwab et al., 2022b). One mid-2020 case-control study in 1,027 patients in Iran showed no change in Covid- 19 infection in endometriosis patients (Moazzami et al., 2021), whilst another smaller case-control study of 401 patients in Italy conducted between March 2020 and April 2021 showed a slight increase in number of infections in endometriosis patients (Barretta et al., 2022). The other studies all used surveys with focuses ranging from gynaecologists’ practice (Uccella et al., 2021), to patients’ anxiety (Arena et al., 2021), pain and disability (Schwab et al., 2021), cannabis use (Armour et al., 2022), opinions on remote care (Rosielle et al., 2021), or patient experience more generally (Demetriou et al., 2021; Evans et al., 2021; Yalçın Bahat et al., 2020). These surveys generally highlight negative impacts of the pandemic on endometriosis patients. In the largest study, including 6,729 responses, 80.7% of endometriosis patients reported a negative impact on their care (Demetriou et al., 2021).

As the pandemic approaches its third year, it is increasingly important to understand endometriosis patients’ experiences to provide better support and allocate health resources. None of the above surveys (Arena et al., 2021; Armour et al., 2022; Ashkenazi et al., 2022; Demetriou et al., 2021; Evans et al., 2021; Nicolás et al., 2022; Rosielle et al., 2021; Schwab et al., 2022a, 2022b, 2021; Uccella et al., 2021; Yalçın Bahat et al., 2020) include data beyond January 2021. We aimed to better understand the ongoing effects of Covid-19 on endometriosis patients, using an online survey sent in mid-2021.

## Methods

Between 27^th^ August and 15^th^ September 2021, an online survey was circulated via an Endometriosis UK mailing list. The online survey was created and hosted by Pro-Forms (https://Pro-Forms.co.uk). We included questions on demographics, experience of Covid-19 infection, and impact of Covid-19 on endometriosis symptoms and surgery. Respondents were eligible if >18 years old, with a diagnosis of endometriosis by a medical professional, and if they were able to understand and respond to questions in English. There were no exclusion criteria otherwise.

The survey was voluntary and circulated by mailing list. Endometriosis UK has approximately 20,000 members, but the survey may have been shared more widely as no password protection or similar was required to enter. No incentives to complete the survey were offered. An HRA- approved participant information sheet and written text at the beginning of the survey gave information to patients, including the names of investigators, the purpose of and likely completion time of the survey (10-15 minutes). Patients gave implied consent by completing the survey, but no other formal written consent was taken. Data were collected and stored on Pro-Forms which adheres to GDPR guidelines and analysed on password-protected computers. No patient identifiable data was collected. The survey was developed on Pro-Forms and was tested by investigators before use. No randomisation or alternation in questions occurred. Adaptive questioning was used - for example in sections asking about Covid-19 hospitalisation. With adaptive questioning, there was a maximum of 39 questions over 3 pages of questions. Only complete surveys could be submitted, with checking an inbuilt function of Pro-Forms. There was no additional step where participants reviewed completed answers before submission. To avoid collection of patient identifiable data, respondents did not need to register, and no IP address recording or cookies were used. View rates and recruitment rates were not measured.

Survey results were analysed using Pro-Forms and Microsoft Excel. No statistical comparisons were made since no direct comparator groups were available. Distribution of continuous variables were assessed by plotting histograms. Continuous data were not normally distributed so are presented as a median with interquartile range in brackets. Numbers of categorical responses are presented as percentages with raw frequency of responses in brackets. No weighting of items or propensity scores were used.

This study received HRA and HCRW research ethic committee approval (REC reference 21/ WM/0062), with initial approval on the 9th April 2021. The study was sponsored by the Royal Free London NHS Foundation Trust. No funding was received for this study. Endometriosis UK provided us with feedback during the development of survey questions and circulated the study to respondents.

## Results

We received 1,089 completed survey responses. Demographics of respondents are shown in Table I. Patients had a median age of 34 and median time since initial endometriosis diagnosis of 4 years. 82.0% (n=893) of respondents were white British, with far fewer respondents of other ethnicities. 2.3% (n=23) of respondents were pregnant at the time of response. 77.8% (n=847) were diagnosed with endometriosis through a prior surgery.

**Table I:** Demographics of patients, based on their survey responses. Continuous variables are presented as median (IQR). Proportions are presented as percentages (raw values in brackets).

Age (years)	34 (28-40)
Time since diagnosis (years)	4 (2-9)
BMI (kg/m2)	26.8 (23.0-31.6)
Ethnic origin	
Asian - Banglageshi	0.2% (2)
Asian - Chinese	0.1% (1)
Asian - Indian	1.4% (15)
Asian - Pakistani	0.6% (6)
Asian - Other	0.5% (5)
Black - African	0.6% (6)
Black - Caribbean	0.6% (6)
Mixed White/Asian	0.8% (9)
Mixed White/Black African	0.1% (1)
Mixed White/Black Caribbean	0.6% (7)
Mixed - Other	1.0% (11)
White - British	82.0% (893)
White - Irish	3.1% (34)
White - Other	7.8% (85)
Other	0.3% (3)
Prefer not to say	0.5% (5)
Smoking status	
Never smoked	62.6% (682)
Ex-smoker	26.6% (290)
Smoker	10.7% (117)
Pregnancy status at time of response	
Pregnant	2.1% (23)
Gestation if pregnant (weeks)	18 (10-22)
Not pregnant	97.2% (1058)
Uncertain	0.7% (8)
Endometriosis Diagnosis	
Surgical diagnosis	77.8% (847)
Imaging finding	13% (142)
Symptoms alone	6.3% (69)
Other	2.8% (31)
Number of prior endometriosis operations	
None	13% (142)
1	40% (436)
2	23.7% (258)
≥3	23.2% (253)
Medical endometriosis treatment prior to pandemic	
None	24.2% (263)
Analgesia	31.4% (342)
Hormonal treatments	35.9% (391)
Other	8.5% (93)
Endometriosis location	
Pelvis	21.5% (686)
Uterus	17.2% (551)
Ovary	20.9% (669)
Bowel	17% (544)
Bladder	11.1% (354)
Diaphragm	1.9% (60)
Thoracic endometriosis	0.9% (28)
Other	5.5% (175)
Not sure	4% (128)
Comorbidities	
Heart disease	0.5% (11)
Kidney disease	0.3% (6)
Hypertension	2.2% (44)
Hypercholesterolaemia	0.9% (18)
Diabetes	0.7% (15)
Thyroid disease	3.6% (73)
Respiratory disease	8.5% (171)
Mental health conditions	25.2% (508)
Other gynaecological conditions	17.9% (360)
Irritable bowel syndrome (IBS)	16% (323)
Coeliac disease	1.1% (22)
Other	11.8% (237)
None	10% (201)
Not Answered	1.2% (24)

*e.g., obstruction of ureter

In terms of the clinical history of respondents’ endometriosis, only 13% (n=142) had never had a prior endometriosis operation whilst 23.2% (n=253) had ≥3 prior endometriosis operations (Table I). 75.8% (n=826) of respondents were on medical treatment for endometriosis, most commonly hormonal treatments (35.9%, n=391). The main anatomical sites of endometriosis reported were pelvis (21.5%, n=686), uterus (17.2%, n=551), ovary (20.9%, n=669) and bowel (17%, n=544). 88.2% (n=864) of patients reported suffering from a medical comorbidity other than endometriosis, of which mental health conditions (25.2%, n=508), other gynaecological conditions (17.9%, n=360) and irritable bowel syndrome (16%, n=323) were most common.

Table II highlights respondents’ experience of Covid-19 infection. 18.8% (n=205) of respondents reported a previous positive Covid-19 PCR test, while a larger 36.2% (n=394) of respondents reported previous Covid-19 symptoms, possibly accounted for by test unavailability in the earlier stages of the pandemic. 1.8% (n=20) of respondents reported being admitted to hospital, with 1 patient reporting intubation and intensive care. We cannot draw any comparisons in terms of Covid-19 risk from this subjective study with multiple biases, but simply to put these numbers into context at this stage of the pandemic, on 15th September 2021 (the last day of our survey), in a total estimated UK population of 67.1 million, cumulatively 7.52 million people (~11% of the total population) had tested positive for Covid-19 and 538,400 patients (0.8% of the population) had been admitted to hospital. 84.6% (n=921) of respondents had been double vaccinated at the time of response, compared to 44.2 million people (~66% of the total population) on 15th September 2021. Most patients (54.7%, n=596) did not feel at high risk of contracting Covid-19 due to their endometriosis and did not believe they were at higher risk of severe Covid-19 due to their endometriosis (57.4%, n=625). 15.9% (n=173) of respondents shielded during the pandemic due to a medical problem they have. Notably, 28 respondents reported having thoracic endometriosis; of these, 6 reported a previous positive PCR test, and none were admitted to hospital.

**Table II:** Survey responses to questions regarding how respondents were affected by Covid-19 infection and the pandemic - including Covid-19 symptoms, testing, vaccination, shielding and hospital admission due to Covid-19. Response proportions are presented as percentages of total survey responses (n=1089) with raw numbers in brackets.

Do you think you have had symptoms of Covid-19 at any point during the pandemic?	
Yes	36.2% (394)
No	51.4% (560)
Maybe/Don’t know	12.4% (135)
If you have experienced symptoms of Covid-19, what symptoms did you experience?	
Fever	8.0% (273)
Cough	8.8% (301)
Sore throat	8.4% (286)
Change or loss of sense of smell	5.9% (203)
Change or loss of sense of taste	6.0% (205)
Weakness or aches and pains	10.0% (343)
Runny or stuffy nose	5.6% (191)
Diarrhoea	4.0% (136)
Shortness of breath	5.9% (202)
Chest pain	5.0% (171)
Abdominal pain	4.4% (152)
Headaches	9.2% (316)
Other	0.7% (24)
Not Answered	18.0% (616)
Have you ever had a PCR swab test for Covid-19?	
Yes	81.2% (884)
No	18.2% (198)
Not sure	0.6% (7)
If you have had a PCR swab test, have you ever tested positive for Covid-19?	
Yes	18.8% (205)
No	62.3% (678)
Not sure	0.2% (2)
Not Answered	18.7% (204)
Have you had an antibody test for Covid-19?	
Yes	22.4% (244)
No	75.0% (817)
Not sure/Don’t know	2.6% (28)
If you have had an antibody test for Covid-19, what was the result?	
Positive	5.6% (61)
Negative	17.3% (188)
Not Answered	77.1% (840)
Have you received a Covid-19 vaccine?	
Yes, one dose	6.2% (67)
Yes, two doses	84.6% (921)
No	9.3% (101)
Do you feel at higher risk of contracting Covid-19 infection because of your endometriosis?	
Yes	16.9% (184)
No	54.7% (596)
Not sure/Don’t know	28.4% (309)
Do you feel at higher risk of having severe Covid-19 (ie needing hospital/oxygen) infection if you were to/have contracted the infection, because of your endometriosis?	
Yes	14.4% (157)
No	57.4% (625)
Not sure/Don’t know	28.2% (307)
Have you ever shielded during the coronavirus pandemic (to protect yourself from infection)?	
Yes	23.3% (254)
No	76.7% (835)
If you did shield, why was this?	
Due to a medical problem that I have	15.9% (173)
Because of someone I live with/am close having a medical problem	10.7% (116)
Not Answered	73.5% (800)
Have you ever been admitted to hospital due to Covid-19?	
Yes	1.8% (20)
No	98.2% (1069)
If you were admitted to hospital, were you admitted to an intensive care unit?	
Yes	0.1% (1)
No	1.8% (20)
Not Sure/Don't know	0.2% (2)
Not Answered	97.9% (1066)
If you were admitted to an intensive care unit, did you require intubation?	
Yes	0.1% (1)
No	0% (0)
Not Sure/Don't know	0% (0)
Not Answered	99.9% (1088)
Did you require oxygen in hospital, if you were admitted?	
Yes	0.6% (7)
No	1.2% (13)
Not Sure/Don't know	0% (0)
Not Answered	98.2% (1069)
Hospital treatments for Covid-19 reported	
Antibiotics	1.1% (12)
Steroids	0.4% (4)
Blood thinning medications	0.3% (3)
Intravenous drips/fluids	0.6% (7)
Special oxygen support (ie ‘non invasive ventilation’, CPAP or BiPAP)	0.3% (3)
Invasive help with feeding - ie an NG tube (down the nose) or TPN (feeding through a vein)	0.1% (1)
Dialysis or filtration of the blood	0% (0)
Special immune therapies eg remdesivir or hydroxycholo- roquine - usually on clinical trials in the UK	0% (0)
Blood transfusion	0% (0)
A urinary catheter	0.2% (2)
Not Answered	97.1% (1072)

When asked to compare endometriosis symptoms during the pandemic to before, large numbers of patients reported worsening of symptoms including pain in general (55.2%, n=601), dysmenorrhea (50.8%, n=553), dyspareunia (31.4%, n=342), dysuria or dyschezia (40.2%, n=438) and menstrual bleeding (36.4%, n=396; Table III). Similar numbers reported that these symptoms were unchanged. Strikingly, 69.2% (n=754) of respondents reported worsening of associated mental health symptoms during the pandemic. There was some discrepancy in the number of people who described worsening of endometriosis symptoms with Covid-19 infection (~500 responses per question) and the number who reported experiencing Covid-19 infection earlier in the survey (205 patients reported a positive Covid- 19 PCR and 394 patients reporting Covid-19 symptoms: Tables II-III). Proportions of respondents who did answer these questions and reported that endometriosis symptoms were either unchanged or worsened by Covid-19 infection were like those whose symptoms were affected overall by the pandemic (Table III).

**Table III:** Survey responses as to effect of Covid-19 pandemic and infection on endometriosis symptoms. Data are presented as percentage of total survey responses (n=1089) with raw numbers in brackets.

During the pandemic period, how would you compare your endometriosis symptoms to before the pandemic?						
Pelvic pain in general	Better	Unchanged	Worse	Not sure	Not a symptom I experience	Not answered
	2.8% (30)	38.0% (414)	55.2% (601)	2.2% (24)	1.6% (17)	0.3% (3)
Menstrual bleeding	Better	Unchanged	Worse	Not sure	Not a symptom I experience	Not answered
	3.8% (41)	41.0% (447)	36.4% (396)	3.8% (41)	14.9% (162)	0.2% (2)
Pelvic pain during your period	Better	Unchanged	Worse	Not sure	Not a symptom I experience	Not answered
	2.3% (25)	34.3% (374)	50.8% (553)	2.2% (24)	10.1% (110)	0.3% (3)
Pain during sexual intercourse	Better	Unchanged	Worse	Not sure	Not a symptom I experience	Not answered
	1.8% (20)	43.2% (470)	31.4% (342)	9.2% (100)	13.9% (151)	0.6% (6)
Pain when urinating or defaecating	Better	Unchanged	Worse	Not sure	Not a symptom I experience	Not answered
	2.2% (24)	40.3% (439)	40.2% (438)	3.5% (38)	13.3% (145)	0.5% (5)
Depression/Anxiety/Mental health	Better	Unchanged	Worse	Not sure	Not a symptom I experience	Not answered
	2.6% (28)	18.1% (197)	69.2% (754)	3.9% (43)	5.9% (64)	0.3% (3)
If you did experience Covid-19 infection, how were your endometriosis symptoms affected compared to before the infection?						
Pelvic pain in general	Better	Unchanged	Worse	Not sure	Not a symptom I experience	Not answered
	0.6% (6)	14.0% (153)	12.9% (140)	6.0% (65)	16.2% (176)	50.4% (549)
Menstrual bleeding	Better	Unchanged	Worse	Not sure	Not a symptom I experience	Not answered
	0.8% (9)	14.4% (157)	9.7% (106)	5.4% (59)	17.7% (193)	51.9% (565)
Pelvic pain during your period	Better	Unchanged	Worse	Not sure	Not a symptom I experience	Not answered
	0.5% (5)	13.5% (147)	12.3% (134)	5.4% (59)	15.7% (171)	52.6% (573)
Pain during sexual intercourse	Better	Unchanged	Worse	Not sure	Not a symptom I experience	Not answered
	0.3% (3)	15.2% (165)	8.3% (90)	6.6% (72)	16.8% (183)	52.9% (576)
Pain when urinating or defaecating	Better	Unchanged	Worse	Not sure	Not a symptom I experience	Not answered
	0.2% (2)	14.6% (159)	9.6% (105)	6.1% (66)	16.3% (178)	53.2% (579)
Depression/Anxiety/Mental health	Better	Unchanged	Worse	Not sure	Not a symptom I experience	Not answered
	0.4% (4)	8.2% (89)	19.7% (215)	4.1% (45)	14.4% (157)	53.2% (579)

Whilst 44% (n=479) of respondents had elective endometriosis surgery planned prior to the pandemic, the majority of these were disrupted (Table IV). 18.7% (n=204) of total respondents did not have a new elective surgery date at the time of response. 5.4% (n=59) of patients required emergency endometriosis-related surgery during the pandemic.

## Discussion

### Main findings

In this study, we highlight ongoing impacts of Covid-19 on UK endometriosis patients. Studies conducted during the first 6 months of the pandemic have highlighted various negative impacts of the pandemic on endometriosis patients (Arena et al., 2021; Armour et al., 2022; Demetriou et al., 2021; Evans et al., 2021; Rosielle et al., 2021; Schwab et al., 2021; Uccella et al., 2021; Yalçın Bahat et al., 2020). This work was conducted when gynaecology services had adapted - with resumption of elective surgeries (Iqbal et al., 2021; Søreide et al., 2020), use of virtual consultations, and national frameworks for restarting non-urgent gynaecology care (RCOG, 2021). Despite this, large numbers of patients (31.4-55.2%) reported worsening of their endometriosis symptoms (Table III). The pattern of Covid-19 infection and symptoms in respondents did not appear different to the general population (Table II), suggesting other pandemic factors are to blame.

Strikingly, 69.2% (n=754) of patients reported worsening of associated mental health symptoms, in keeping with several studies which highlighted impacts of the pandemic on endometriosis patients’ mental health (Arena et al., 2021; Demetriou et al., 2021; Evans et al., 2021; Yalçın Bahat et al., 2020). Clearly there have been significant negative effects on the general population’s mental health (Pierce et al., 2020). However, given that endometriosis is tightly associated with mental health issues, mental health impacts perception of endometriosis symptoms, and concurrently both impact patients’ quality of life (Facchin et al., 2015; Laganà et al., 2017), this is a significant aspect that may continue to be overlooked by gynaecologists.

There is no evidence that endometriosis increases patients’ vulnerability to Covid-19 infection, and the largest case-control study so far found no increased risk (Moazzami et al., 2021). Given the nature of our study, we cannot evaluate risk of Covid-19 in our cohort. Nevertheless, it is interesting to highlight those 28 respondents reported thoracic endometriosis, which could theoretically increase risk of severe Covid-19 infection, though did not seem to have an effect in our cohort.

Low numbers of patients felt at higher risk of contracting Covid-19 (16.9%, n=184) or severe Covid-19 infection (14.4%, n=157; Table II). A previous study found that 54.2% of respondents felt more vulnerable to Covid-19 (Demetriou et al., 2021). This may reflect a change in attitudes of patients as the pandemic has progressed. Interestingly, 23.3% (n=254) of our respondents shielded during the pandemic, with 15.9% (n=173) saying this was due to a medical problem they have. We did not directly ask whether this was because of endometriosis to avoid controversy. This large number of respondents shielded, despite endometriosis patients not being defined as clinically extremely vulnerable by the UK government. Vaccine uptake in respondents was high, with 84.6% (n=921) receiving 2 doses of vaccine at the time of response.

Endometriosis operations were significantly disrupted during the pandemic. A high proportion of respondents (44%, n=479) were planned for elective surgery during the pandemic. Only 7.6% of total respondents (n=83) reported this was unaffected. Importantly, 18.7% (n=204) reported that they did not have a new date for surgery. There is a large NHS backlog, with cancer services prioritised. Within endometriosis, national frameworks recommend prioritising patients with pelvic pain refractory to medical treatments and endometriosis causing ureteric or bowel obstruction (RCOG, 2021). This prioritisation system is simplistic and does not consider impacts on patients’ quality of life, reproductive capabilities, and wider economic impact. Many patients with ‘less severe’ endometriosis could be left waiting for long periods of time. Before the pandemic, a parliamentary group found that 30% of patients waited >6 months for surgery, with an average length to diagnosis of 8 years (Endometriosis APPG, 2021). These waits will only have increased.

### Strengths and Limitations

This studies’ strengths are its size and timing. To our knowledge, this is the third largest study of endometriosis patients during the Covid-19 pandemic and the largest study of UK-only patients. No studies have examined endometriosis patients’ views past the 1-year stage of the pandemic.

There are obvious limitations to our study given its nature, including selection bias, as it was circulated by a charity providing support to disease sufferers. We did not strictly limit survey access by password protection or IP address registration, or by more directly reaching out to known NHS patients, so there are unknowns in how the public may have circulated the survey link more widely, or who may have answered the survey. Our respondents were young, predominantly white British, and had relatively severe endometriosis (with high numbers requiring surgical management). Our cohort therefore may not be truly representative of the UK, and clearly under-represents BAME groups. We did not assess locations of respondents within the UK, or their financial status, and did not ask whether they were accessing private care. Moreover, those more affected by the pandemic may have been more likely to respond, exaggerating effects. There were inconsistencies in survey completion, with discrepancies in numbers reporting symptom changes due to Covid-19 infection and those who reported actual Covid-19 infection. Particular ways in which patients’ symptoms have changed were not examined in depth - for example, we used the term ‘menstrual bleeding’ in our survey, which is physiological not pathological, but could have been interpreted by respondents to mean heavy bleeding during their period, irregular bleeding, or otherwise. Nevertheless, we believe this study succeeds in highlighting that endometriosis patients have been significantly affected by the Covid-19 pandemic and that continued work is needed to optimise treatment strategies and resource allocation.

### Interpretation

Our findings are consistent with previous studies, which highlighted that endometriosis patients continue to be affected by the pandemic, despite not appearing to be directly at risk of more severe Covid-19 infection, and despite adaptation of gynaecology services. Mental health symptoms and surgical management of endometriosis were particularly affected. Further research into how gynaecologists could better support patients whilst waiting for surgery should be a priority. Improved holistic care including a focus on mental health symptoms within multidisciplinary teams could improve patients’ quality of life.

## Conclusion

Research into the effects of Covid-19 on endometriosis so far has been limited - with only 14 other published research studies of surveys and small case-control studies. Though endometriosis rarely requires emergency treatment, and generally affects a younger population, with no current link to more severe Covid-19 infection, this early data highlights a strikingly worsened symptom burden, particularly of associated mental health symptoms, during the pandemic. In addition, elective surgery has been significantly disrupted. Greater attention in terms of healthcare provision, research and support is required for endometriosis patients as the pandemic continues.
